# Supplementary figures and images for: A Pilot Study of Methods for Evaluating the Effects of Arousal and Emotional Valence on Performance of Racing Greyhounds
Source: Animals (Basel). 2020 Jun 15;10(6):1037. doi: 10.3390/ani10061037 (PMC7341205; doi:10.3390/ani10061037)

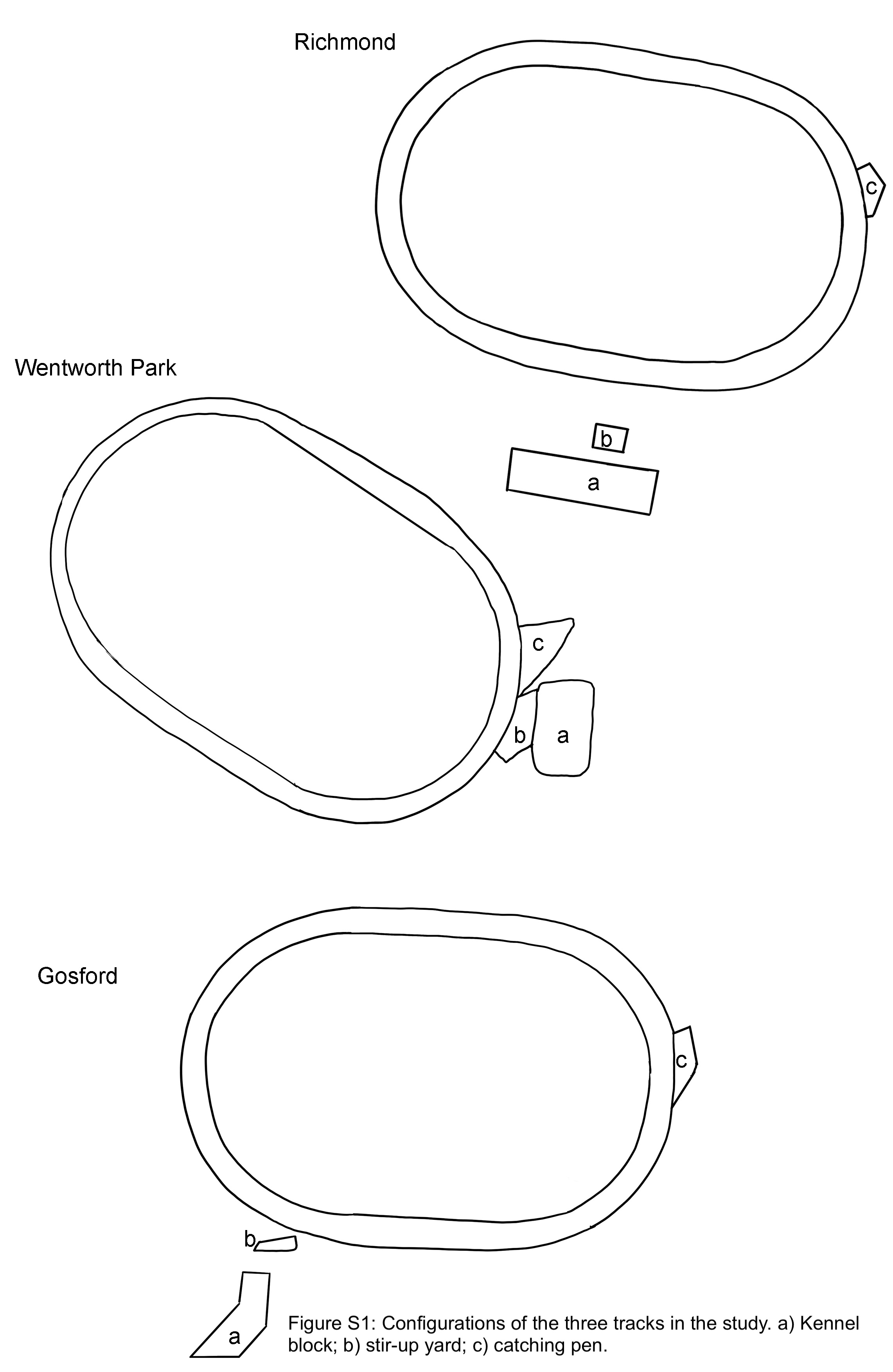

Supplement: Supplementary file 1 [file animals-10-01037-s001.jpg]
